# Validation of Vancomycin Area under the Concentration—Time Curve Estimation by the Bayesian Approach Using One-Point Samples for Predicting Clinical Outcomes in Patients with Methicillin-Resistant *Staphylococcus aureus* Infections

**DOI:** 10.3390/antibiotics11010096

**Published:** 2022-01-13

**Authors:** Takashi Ueda, Yoshio Takesue, Kazuhiko Nakajima, Kaoru Ichiki, Kaori Ishikawa, Kumiko Yamada, Toshie Tsuchida, Naruhito Otani, Yoshiko Takahashi, Mika Ishihara, Shingo Takubo, Hiroki Ikeuchi, Motoi Uchino, Toshimi Kimura, Kazuaki Matsumoto, Kazutaka Oda, Takeshi Kimura

**Affiliations:** 1 Department of Infection Control and Prevention, Hyogo College of Medicine, 1-1, Mukogawa-cho, Nishinomiya 663-8501, Japan; takesuey@hyo-med.ac.jp (Y.T.); nakajima@hyo-med.ac.jp (K.N.); ichiki@hyo-med.ac.jp (K.I.); i-kaori@hyo-med.ac.jp (K.I.); yamakumi@hyo-med.ac.jp (K.Y.); tsuchida@huhs.ac.jp (T.T.); 2 Department of Clinical Infectious Diseases, Tokoname City Hospital, 3-3-3 Asukadai, Tokoname 479-8510, Japan; 3 Department of Public Health, Hyogo College of Medicine, 1-1, Mukogawa-cho, Nishinomiya 663-8501, Japan; n-otani@hyo-med.ac.jp; 4 Department of Pharmacy, Hyogo College of Medicine Hospital, 1-1, Mukogawa-cho, Nishinomiya 663-8501, Japan; yktabu@hyo-med.ac.jp (Y.T.); ykkura@hyo-med.ac.jp (M.I.); stakubo@hyo-med.ac.jp (S.T.); t-kimura@hyo-med.ac.jp (T.K.); 5 Department of Surgery, Hyogo College of Medicine, 1-1, Mukogawa-cho, Nishinomiya 663-8501, Japan; ikeuci2s@hyo-med.ac.jp (H.I.); uchino2s@hyo-med.ac.jp (M.U.); 6 Department of Pharmacy, Tokyo Women’s Medical University Hospital, 8-1, Kawada-cho, Shinjuku-ku, Tokyo 162-0054, Japan; kimura.toshimi@twmu.ac.jp; 7 Division of Pharmacodynamics, Faculty of Pharmacy, Keio University, 1-5-30 Shibakoen, Minato-ku, Tokyo 105-8512, Japan; matsumoto-kz@pha.keio.ac.jp; 8 Department of Pharmacy, Kumamoto University Hospital, 1-1-1, Honjo, Chuo-ku, Kumamoto 860-8556, Japan; kazutakaoda@kuh.kumamoto-u.ac.jp

**Keywords:** vancomycin, area under the concentration–time curve, Bayesian estimation, methicillin-resistant *Staphylococcus aureus* infection, nephrotoxicity

## Abstract

Area under the concentration–time curve (AUC)-guided vancomycin treatment is associated with decreased nephrotoxicity. It is preferable to obtain two samples to estimate the AUC. This study examined the usefulness of AUC estimation via trough concentration (C_min_)-only sampling of 260 adults infected with methicillin-resistant *Staphylococcus aureus* (MRSA) who received vancomycin. The exact C_min_ sampling time was used for Bayesian estimation. A significantly higher early treatment response was observed in patients with a day 2 AUC ≥ 400 µg·h/mL than those with <400 µg·h/mL, and a significantly higher early nephrotoxicity rate was observed in patients with a day 2 AUC ≥ 600 µg·h/mL than those with <600 µg·h/mL. These AUC cutoff values constituted independent factors for each outcome. In sub-analysis, the discrimination ability for early clinical outcomes using these AUC cutoffs was confirmed only in patients with q12 vancomycin administration. A significant difference in early treatment response using the 400 µg·h/mL cutoff was obtained only in patients with low-risk infections. The usefulness of the vancomycin AUC target to decrease nephrotoxicity while assuring clinical efficacy was even confirmed with a single C_min_ measurement. However, assessment with two samples might be required in patients with q24 administration or high/moderate-risk MRSA infections.

## 1. Introduction

Vancomycin remains a first-line therapy for methicillin-resistant *Staphylococcus aureus* (MRSA) infections [1]. The ratio of the area under the concentration–time curve (AUC) over 24 h to the minimum inhibitory concentration (MIC) has been demonstrated to reflect the maximal clinical effects of vancomycin [2,3,4,5]. The trough concentration (C_min_) has been used as a surrogate marker of the AUC. However, Neely et al. [6] found that among patients with an AUC of ≥400 µg·h/mL and an organism vancomycin MIC of 1 μg/mL, approximately 60% were expected to have a C_min_ < 15 μg/mL. Thus, C_min_-guided dosing targeting a C_min_ ≥ 15 μg/mL may lead to excessive vancomycin exposure. Phillips [7] reported that determining vancomycin exposure by computation of the AUC may help to truly individualize therapy and better manage toxicity risk, rather than relying on C_min_ alone. A revised consensus guideline recommended AUC-guided dosing with the use of Bayesian methods [4]. Tsutsuura et al. [8] conducted a systematic review and meta-analysis and demonstrated that the incidence of nephrotoxicity tended to be lower in AUC-guided monitoring than in C_min_-guided monitoring. In addition, they reported that a high AUC/MIC ratio (cutoff, 400 ± 15%) was associated with a significantly lower treatment failure rate, and a high AUC (cutoff, 600 ± 15%) significantly increased the risk of nephrotoxicity [8].

Although it has been considered preferable to obtain two pharmacokinetic (PK) samples to accurately estimate the AUC using the Bayesian approach, updated therapeutic drug monitoring (TDM) guidelines [4] suggested that the C_min_ alone may be sufficient to estimate the AUC in some patients. For the wide distribution of AUC-guided dosing in clinical practice, further validation of an AUC estimation using only the C_min_ is required. When a population PK model based on richly sampled data is used as a Bayesian prior, C_min_-only data can be used to generate accurate AUC estimates. In contrast, a population PK model based on limited samples was worse at predicting the true AUC from C_min_-only data, and two-point measurements may be required as a better Bayesian prior [6]. Previously, we studied the performance of AUC estimation with a population PK model based on two-point samples compared with the reference AUC calculated according to the log-linear trapezoidal rule using eight measured drug concentrations after a single intravenous infusion [9]. The AUC estimation using two concentrations produced the least bias in patients with vancomycin q12h administration. By contrast, the AUC estimation using only the C_min_ produced moderate and unignorable bias in patients with vancomycin q12h and q24h administration, respectively. Considering the decrease in accuracy, we suspected that AUC estimation using only the trough concentration might be avoided in patients with difficult-to-treat MRSA infections and in patients with kidney dysfunction who are likely to be prescribed once daily dosing.

The primary purpose of this study was to confirm the correlation between clinical outcomes and pre-described AUC cutoff levels (increase of treatment success: ≥400 µg·h/mL; decreased risk for nephrotoxicity < 600 µg·h/mL), using AUC estimation by one-point sampling. As a subgroup-analysis, the relationship was separately evaluated in patients with vancomycin q12h and q24h administration, and those with high/moderate-risk and low-risk MRSA infections. If a clear correlation was obtained only in patients with vancomycin q12h administration or in patients with low-risk MRSA infections, the result might support previously mentioned hypotheses.

## 2. Results

### 2.1. Patient Characteristics

Among 444 patients with MRSA infections, 312 patients met the inclusion criteria (reasons for exclusion: less than 18 years (33), intermittent hemodialysis (71), and continuous renal replacement therapy (28)), but 52 of these patients were excluded for the following reasons: pregnancy (6), the previous use of antimicrobial agents with anti-MRSA activity (31), and MRSA infections with a vancomycin MIC = 2 mg/L (15). Thus, 260 patients were included in the study. Vancomycin was administered with q12h in 202 patients and q24h in 58 patients, and vancomycin was used in 105 patients with high/moderate-risk infections and in the remaining 155 patients with low-risk infections.

The baseline demographics of the enrolled patients are presented in Supplementary Table S1, and 17.3% of patients required an ICU stay while 35.4% had Acute Physiology and Chronic Health Evaluation (APACHE) II scores >10. Twice daily administration was employed in 202 patients with eGFR ≥ 70 mL/min/1.73 m^2^, and no patients with eGFR < 70 mL/min/1.73 m^2^. Once daily administration was employed in 10 patients with eGFR ≥ 70 mL/min/1.73 m^2^ and 48 patients with eGFR < 70 mL/min/1.73 m^2^. For 202 patients with vancomycin q12h administration, 69, 64, and 34 patients were treated using regimens A, B, and C, respectively. A loading dose was used in 98 patients (48.5%; 30 mg/kg (28) and 25 mg/kg (70)), and the maintenance doses were 17.5–22.5 mg/kg twice daily in 54 patients, 12.5–17.5 mg/kg twice daily in 133 patients, and 7.5–12.5 mg/kg twice daily in 15 patients.

### 2.2. Bayesian Estimation of the AUC and the C_min_ on Day 1, Day 2, and the Steady-State

**Figure 1:**
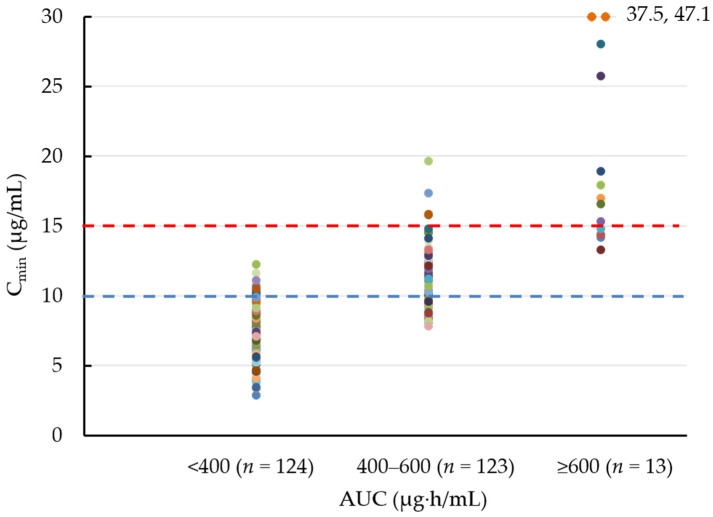
C_min_ distribution in each AUC category on day 2.

The exact sampling times after the previous dose in the initial TDM were <9 (*n* = 0), 9–10 (*n* = 12), 10–11 (*n* = 46), 11–12 (*n* = 111), 12–13 (*n* = 29), and ≥13 h (*n* = 4) in patients with vancomycin q12h administration and were <20 (*n* = 0), 20–21 (*n* = 4), 21–22 (*n* = 10), 22–23 (*n* = 16), 23–24 (*n* = 24), 24–25 (*n* = 3), and ≥25 h (*n* = 1) in patients with vancomycin q24h administration. The median AUC values of day 1, day 2, and the steady-state for the initial vancomycin regimen AUC were 382.7, 403.9, and 422.4, respectively (Table 1), while the median C_min_ values were 8.0, 9.8, and 10.3 μg/mL, respectively. The C_min_ distribution in each AUC category is presented in Figure 1. The median C_min_ was 7.4 μg/mL for AUC < 400 µg·h/mL, 11.0 μg/mL for AUC = 400–600 µg·h/mL, and 17.0 μg/mL for AUC ≥ 600 µg·h/mL. Among 123 patients who achieved the AUC target of 400–600 µg·h/mL, only four patients (3.3%) had the previously recommended C_min_ target of 15–20 μg/mL [10], suggesting that C_min_ is far from an adequate surrogate maker of AUC.

### 2.3. Early Treatment Response and Early Nephrotoxicity

**Figure 2:**
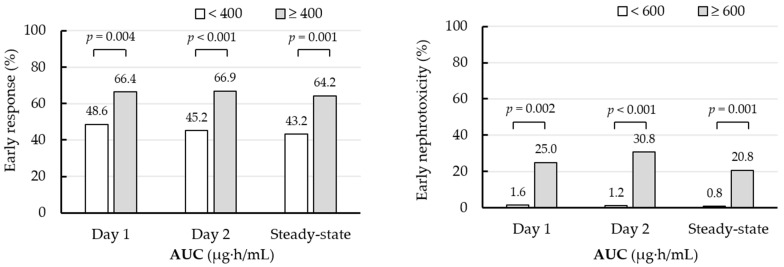
Early clinical response and early nephrotoxicity rates after 48–72 h of vancomycin administration according to the AUC on days 1–2 and at steady-state.

Figure 2 shows the early treatment response and early nephrotoxicity according to each AUC cutoff value on day 2. An early treatment response and treatment success at the end of vancomycin therapy (EOT) were obtained in 147 of 260 patients (56.5%) and 180 of 238 patients (75.6%), respectively. The mortality rate at 28 days was 6.2% (16/260 patients). Early nephrotoxicity and nephrotoxicity during vancomycin therapy were observed in seven of 260 patients (2.7%) and 35 of 260 patients (13.5%), respectively. A significantly higher early clinical response rate was obtained in patients with a day 2 AUC ≥ 400 µg·h/mL than those with <400 µg·h/mL (66.9% versus 45.2%, *p* < 0.001). In regard to safety, a significantly higher early nephrotoxicity rate was observed in patients with a day 2 AUC ≥ 600 µg·h/mL than those with <600 µg·h/mL (30.8% versus 1.2%, *p* < 0.001).

In multivariate analysis, a day 2 AUC ≥ 400 µg·h/mL was one of the independent factors associated with an early clinical response (OR = 2.02, 95% CI = 1.15–3.53, *p* = 0.014). The risk factors for a decreased early treatment response were collagen disease (OR = 0.28, 95% CI = 0.12–0.63, *p* = 0.002), intensive care unit stay (OR = 0.34, 95% CI = 0.15–0.78, *p* = 0.011), and an APACHE II score >10 (OR = 0.44, 95% CI = 0.23–0.84, *p* = 0.014) (Table 2). In addition, a day 2 AUC ≥ 600 µg·h/mL (OR = 44.77, 95% CI = 6.65–301.65, *p* < 0.001) and concomitant piperacillin/tazobactam use (OR 12.93, 95% CI = 1.87–89.49, *p* = 0.010) were independent factors for early nephrotoxicity (Table 3).

### 2.4. Sub-Analyses of Clinical Outcomes According to Day 2 AUC Cutoff Values in Patients with Vancomycin q12h and q24h Administration, and Those with High/Moderate-Risk and Low-Risk MRSA Infections

Significant discrimination ability based on each day 2 AUC cutoff value for early treatment response (*p* = 0.001) and early nephrotoxicity (*p* < 0.001) was confirmed only in patients with q12 vancomycin administration (Table 4). By contrast, a significant difference in early nephrotoxicity between a day 2 AUC ≥ 600 µg·h/mL and <600 µg·h/mL was observed in both patients with high/moderate-risk and low-risk MRSA infections. However, a significant difference in the early treatment response rate by a cutoff AUC of 400 µg·h/mL was obtained only in patients with low-risk MRSA infections (Table 5).

## 3. Discussion

To improve clinical outcomes, the target vancomycin concentration should be achieved early during the course of therapy. Because Bayesian estimation does not require steady-state serum vancomycin concentrations, it enables the early assessment of AUC target attainment. Casapao et al. [11] reported that higher day 1 exposure resulted in a lower rate of clinical failure and a lower rate of persistent bacteremia in patients with MRSA bacteremia. Lodise et al. [12] suggested that the day 2 AUC should be maintained below approximately 515 µg·h/mL to maximize efficacy and minimize the likelihood of nephrotoxicity. However, in a prospective, multicenter study of adult patients with MRSA bacteremia, a higher day 2 AUC/MIC ratio was not associated with a lower rate of failure but was associated with nephrotoxicity [12]. In our study, a day 2 AUC ≥ 600 µg·h/mL of the initial vancomycin regimen was an independent risk factor for early nephrotoxicity. In addition, a day 2 AUC ≥ 400 µg·h/mL was an independent factor associated with an increased early treatment response. Even with one-point sampling, the usefulness of a target AUC of 400–600 µg·h/mL, which was recommended by recent guidelines [4], was confirmed in our study.

Oda et al. [9] demonstrated that AUC estimation using only the C_min_ produced unignorable bias compared with the reference AUC estimated by multiple samples from patients with vancomycin q24h administration. In our study, significant discrimination ability based on the recommended AUC cutoff value for clinical outcomes was not confirmed in patients with q24h administration. In addition, a significant difference in the early treatment response rate at a cutoff AUC of 400 µg·h/mL was not obtained in patients with high/moderate-risk MRSA infections, and a less biased estimation with a two-point sample might be required for these difficult-to-treat MRSA infections.

Our study had several limitations. First, this study was conducted retrospectively in a single institution. Second, a comparison between AUC estimation using a single C_min_ and that using two-point or multiple samples might be required to verify the AUC-guided dosing by C_min_-only samples. Third, the AUC/MIC ratio was not evaluated in this study. Although patients infected by MRSA strains with a vancomycin MIC = 2 μg/mL were excluded from the study, the separate identification of strains with MICs of 1 and 0.5 μg/mL was not possible in our laboratory report. However, because of the insignificant difference caused by a two-fold dilution in the measurement of the MIC, the AUC/MIC ratio has excessive sensitivity to errors in the MIC, and there are no data to support decreasing the dose to achieve the targeted AUC/MIC ratio of 400–600 if the MIC is less than 1 mg/L. Finally, because clinical outcomes at 48–72 h after the start of therapy were evaluated, nephrotoxicity was analyzed in only seven of 260 patients, and this might have had a significant influence on the results of safety evaluation. If nephrotoxicity was evaluated after reaching a steady state, an increased number of patients would be included in the safety analysis, and 35 patients experienced nephrotoxicity during vancomycin therapy. However, it is imperative to select an exposure that precedes the outcome and is not in the causal pathway.

## 4. Materials and Methods

### 4.1. Patients and Protocol

The study was approved by the Institutional Review Board of Hyogo College of Medicine (No. 3582). The institutional review board waived the requirement for informed consent from patients included in this study. This retrospective study was conducted between April 2011 and May 2020. The study included adult patients who were treated with vancomycin for MRSA infections, who underwent TDM, and who received at least 3 days of vancomycin treatment. The exclusion criteria were as follows: hypersensitivity to vancomycin; pregnancy; age < 18 years, thrice-daily vancomycin administration; intermittent hemodialysis or continuous renal replacement therapy; receipt of any concomitant antibiotics with anti-MRSA activity; receipt of antibiotics with anti-MRSA activity for >24 h within the previous 3 days; receipt of concomitant nephrotoxic antimicrobial agents; and isolation of strains with a vancomycin MIC = 2 μg/mL.

Regimens A–C were adopted in patients with normal renal function (eGFR ≥70), and dosage reduction of each regimen was performed in patients with reduced renal function. Regimen A (15 mg/kg twice daily without a loading dose) was recommended between April 2011 and December 2015. Regimen B (loading dose of 25 mg/kg and maintenance dose of 15 mg twice daily) was provided between January 2016 and May 2018, and regimen C (loading dose of 25–30 mg/kg and maintenance dose of 20 mg/kg twice daily) was administered between June 2018 and May 2020.

Only C_min_-guided dosing was conducted during the study period. The target C_min_ was 10–15 μg/mL during initial TDM, and dosing adjustment to achieve C_min_ 15–20 μg/mL was performed only in patients with complicated MRSA infections or those without clinical responses [13]. An initial C_min_ sample was obtained before the fifth dose. The diagnosis of each type of infection, excluding respiratory tract infection, was based on the definitions of the guidelines issued by the National Healthcare Safety Network (CDC/NHSN surveillance definitions for surgical site infections. Centers for Disease Control and Prevention website: http://www.cdc.gov/nhsn/pdfs/pscmanual/17pscnosinfdef_current.pdf (Published 2018, accessed on 27 August 2020). Infections with at least one of the following signs were analyzed: core temperature > 37.8 °C, total peripheral white blood cell (WBC) count > 10,000/mm^3^, or C-reactive protein (CRP) > 3.0 mg/dL. The presence of pneumonia was identified by chest X-rays or CT scans consistent with pneumonia and at least two of the following signs or symptoms: new-onset or worsening cough; purulent sputum or increased suctioning requirements; auscultatory findings of pneumonia; dyspnea, tachypnea, or respiratory rate ≥ 30 min; hypoxemia; worsening gas exchange; and at least one inflammatory sign [14]. The MIC of vancomycin was measured using microdilution methods in accordance with the Clinical and Laboratory Standards Institute testing guidelines (M02 and M07, 2018) [15].

### 4.2. AUC Evaluation

The vancomycin concentration was measured using a commercial reagent kit (Vanc Flex; Siemens Healthcare Diagnostics, Tokyo, Japan). The coefficient of the dynamic range was 0.8–50 μg/mL (as specified by the manufacturer). The individual PK of vancomycin was retrospectively analyzed using the Bayesian estimation software PAT (Practical AUC-guided TDM for vancomycin) [9]. PAT was developed on the R version 3.6.2/Windows OS 10 system for personal computers and smartphones (sample: https://pharmacokinetic-simulation.shinyapps.io/app-ver1/, accessed on 7 October 2021). The Shiny package, which is an R function for the development of a web application (https://shiny.rstudio.com/, accessed on 7 October 2021), was used as the main user interface, which can actively render the results of calculations from R to the html format in accordance with the input. A previously reported Japanese population PK model [16] was used for the Bayesian estimation. The performance of the AUC estimation by PAT was validated by comparison with the reference AUC calculated according to the log-linear trapezoidal rule [9]. The exact times after the previous dose were used in the Bayesian estimation. Using the one-point serum concentration of the initial vancomycin regimen, the AUC and C_min_ for day 1, day 2, and the steady-state were estimated by the Bayesian software. The cutoff for the AUC value was defined as 400 µg·h/mL for the treatment response and 600 µg·h/mL for nephrotoxicity, and the AUC of day 2 was used to evaluate the relationship with an early clinical response and nephrotoxicity [12].

### 4.3. Adverse Effects and Clinical Efficacy

As an adverse effect, nephrotoxicity was evaluated. Nephrotoxicity was defined as an increase in serum creatinine levels of >0.5 mg/L or 50% versus baseline. The early occurrence of nephrotoxicity at 48–72 h after the start of therapy was evaluated. The rate of nephrotoxicity during vancomycin therapy was also evaluated. An early clinical response at 48–72 h after the start of therapy was investigated. We defined patients as responders if they had a ≥30% decrease in the total peripheral WBC count or CRP level, a reduction in fever (defined as a daily maximum temperature decrease of >0.3 °C for at least 2 consecutive days in febrile patients), no worsening clinical features, and no death within 96 h [17,18]. Treatment success at the EOT was also evaluated and defined as survival with resolution or improvement of all core symptoms and signs of infection to the extent that further antibacterial therapy with anti-MRSA activity was unnecessary. Patients in whom vancomycin was changed to other antimicrobial agents because of adverse effects were excluded from the evaluation of clinical success at the EOT.

### 4.4. Subgroup Analyses

The type of infection or the source of infection are important parameters to evaluate treatment efficacy. Because the population is highly heterogeneous regarding the source of infection, we divided the patients into high/moderate and low-risk infections. High/moderate risk infections consisted of bloodstream infections and complicated infections (endocarditis, ventilator-associated pneumonia, osteomyelitis and arthritis infections, and central nervous system infections) [19]. Low-risk infections included skin and soft tissue infections, urinary tract infections, intra-abdominal infections, and cases of pneumonia with the exception of ventilator-associated pneumonia. Clinical outcomes according to AUC cutoff values were evaluated in patients with high/moderate infections and low-risk MRSA infections. Previously, we reported that AUC estimation using only the C_min_ in patients with vancomycin q24h administration produced unignorable bias compared with the reference AUC based on eight samples [9], and sub-analysis was also performed in patients with vancomycin q12h and q24h administration.

### 4.5. Statistical Analysis

Parametric variables were analyzed using the Student’s *t*-test, whereas nonparametric variables were analyzed using the Mann–Whitney *U*-test or Fisher’s exact test. Multivariate analyses were performed to determine the adjusted odds ratio (OR) for early clinical responses and early nephrotoxicity. Univariate analysis was estimated for each variable with the chi-squared test, and potential confounders were examined via cross-tabulation. Variables selected in univariate analysis (*p* < 0.1) were subsequently entered into a stepwise logistic regression model to estimate the magnitude of association (adjusted OR and 95% confidence interval (CI)). The level of significance was set at *p* < 0.05. SPSS ver. 24 (SPSS Inc., Armonk, NY, USA) was used to perform these analyses.

## 5. Conclusions

The usefulness of the previously reported vancomycin AUC target to decrease nephrotoxicity while assuring clinical efficacy was confirmed with a single C_min_ measurement. However, assessment with a two-point sample might be required in patients with renal dysfunction who are likely to be prescribed once daily dosing or have bacteremia/complicated MRSA infections.

## Figures and Tables

**Table 1:** AUC and C_min_ on days 1–2 and at steady-state.

Pharmacokinetic Parameter		Pharmacokinetic Parameters of the Initial Vancomycin Regimen		
		Day 1	Day 2	Steady-State
AUC (µg·h/mL)	Median (IQR)	382.7 (319.4–455.4)	403.9 (332.4–454.0)	422.4 (351.1–485.8)
	<400	144 (55.4%)	124 (47.7%)	95 (36.5%)
	400–600	104 (40.0%)	123 (47.3%)	141 (54.2%)
	≥600	12 (4.6%)	13 (5.0%)	24 (9.2%)
C_min_ (μg/mL)	Median (IQR)	8.0 (5.9–10.1)	9.8 (7.5–11.3)	10.3 (7.9–12.3)
	<10	191 (73.5%)	140 (53.8%)	121 (46.5%)
	10–15	63 (24.2%)	107 (41.2%)	110 (42.3%)
	15–20	2 (0.8%)	9 (3.5%)	20 (7.7%)
	≥20	4 (1.5%)	4 (1.5%)	9 (3.5%)

C_min_: trough concentration; AUC: area under the concentration–time curve; IQR: interquartile range.

**Table 2:** Univariate and multivariate analyses of the potential variables affecting an early treatment response.

Factors	No. of Patients with an Early Treatment Response (%)		*p* Value by Univariate Analysis	Adjusted Odds Ratio (95% Confidence Interval)	*p* Value by Multivariate Analyses
	Patients with Factor	Patients without Factor			
Day 2 AUC ≥ 400 µg·h/mL	91/136 (66.9%)	56/124 (45.2%)	<0.001	2.02 (1.15–3.53)	0.014
>65 years	75/145 (51.7%)	72/115 (62.6%)	0.079		
Heart disease	37/77 (48.1%)	110/183 (60.1%)	0.073		
Collagen disease	12/35 (34.3%)	135/225 (60.0%)	0.004	0.28 (0.12–0.63)	0.002
Chronic respiratory disease	12/30 (40.0%)	135/230 (58.7%)	0.052		
Serum albumin <2.5 g/dL	38/79 (48.1%)	109/181 (60.2%)	0.070		
Ventilator use	16/54 (29.6%)	131/206 (63.6%)	<0.001		
Intensive care unit stay	12/45 (26.7%)	135/215 (62.8%)	<0.001	0.34 (0.15–0.78)	0.011
APCHE II score >10	34/92 (37.0%)	113/168 (67.3%)	<0.001	0.44 (0.23–0.84)	0.014
Immunosuppressive therapy	2/9 (22.2%)	145/251 (57.8%)	0.043		
VAP	10/38 (26.3%)	137/222 (61.7%)	<0.001		
Skin and soft tissue infection	50/63 (79.4%)	97/197 (49.2%)	<0.001		
Respiratory tract infectionsexcept for VAP	30/68 (44.1%)	117/192 (60.9%)	0.016		

AUC: area under the concentration–time curve; VAP: ventilator-associated pneumonia.

**Table 3:** Variables associated with early nephrotoxicity in univariate and multivariate analyses.

Factors	No of Patients with Early Nephrotoxicity (%)		*p* Value by Univariate Analysis	Adjusted Odds Ratio (95% Confidence Interval)	*p* Value by Multivariate Analyses
	Patients with Factor	Patients without Factor			
Day 2 AUC ≥ 600 µg·h/mL	4/13 (30.8%)	3/247 (1.2%)	<0.001	44.77 (6.65–301.65)	<0.001
Concomitant piperalin/tazobactam	5/54 (9.3%)	2/206 (1.0%)	0.005	12.93 (1.87–89.49)	0.010

AUC: area under the concentration–time curve.

**Table 4:** Early treatment response and early occurrence of nephrotoxicity according to each cutoff AUC on day 2 in patients with vancomycin q12h and q24h administration.

Pharmacokinetics Parameter		Early Treatment Response, No. of Patients (%)			
		q12h Administration		q24h Administration	
a. Early treatment response					
AUC on day 2 (µg·h/mL)	<400	44/92 (47.8%)	reference	12/32 (37.5%)	reference
	≥400	77/110 (70.0%)	*p* = 0.001	14/26 (53.8%)	*p* = 0.213
b. Early nephrotoxicity					
AUC on day 2 (µg·h/mL)	<600	2/189 (1.1%)	reference	1/58 (1.7%)	reference
	≥600	4/13 (30.8%)	*p* < 0.001	0/0	–

AUC: area under the concentration–time curve.

**Table 5:** Early treatment response and early occurrence of nephrotoxicity according to each cutoff AUC on day 2 in patients with high/moderate-risk and low-risk MRSA infections.

Pharmacokinetics Parameter		Early Treatment Response, No. of Patients (%)			
		High/Moderate-Risk MRSA Infections		Low-Risk MRSA Infections	
a. Early treatment response					
AUC on day 2 (µg·h/mL)	<400	19/51 (37.3%)	reference	37/73 (50.7%)	reference
	≥400	29/54 (53.7%)	*p* = 0.091	62/82 (75.6%)	*p* = 0.001
b. Early nephrotoxicity					
AUC on day 2 (µg·h/mL)	<600	1/100 (1.0%)	reference	2/147 (1.4%)	reference
	≥600	2/5 (40.0%)	*p* = 0.005	2/8 (25.0%)	*p* = 0.013

AUC: area under the concentration–time curve.

## Data Availability

The datasets used and/or analyzed during the current study are available from the corresponding author on reasonable request.

## Supplementary Materials

The following supporting information can be downloaded at: https://www.mdpi.com/article/10.3390/antibiotics11010096/s1, Table S1: Base-line demographics of the enrolled patients.
