# Supplementary material for: Validation of Vancomycin Area under the Concentration—Time Curve Estimation by the Bayesian Approach Using One-Point Samples for Predicting Clinical Outcomes in Patients with Methicillin-Resistant Staphylococcus aureus Infections
Source: Antibiotics (Basel). 2022 Jan 13;11(1):96. doi: 10.3390/antibiotics11010096 (PMC8772855; doi:10.3390/antibiotics11010096)
Supplement: Supplementary file 1 [file antibiotics-11-00096-s001.zip › antibiotics-1526412-supplementary.pdf]

antibiotics

Table S1. Baseline demographics of the enrolled patients

	No. of patients (%)				
	Total population (n=260)	High/moderate-risk infections (n=105)	Low-risk infections (n=155)	Vancomycin q12h (n=202)	Vancomycin q24h (n=58)
Male	169 (65.0%)	69 (65.7%)	100 (64.5%)	125 (61.9%)	44 (75.9%)
> 65 years	145 (55.8%)	59 (56.2%)	86 (55.5%)	100 (49.5%)	45 (77.6%)
Body mass index ≥ 25	39 (15.0%)	19 (18.1%)	20 (12.9%)	27 (13.4%)	12 (20.7%)
eGFR < 70 (mL/min/1.73 m^2)	48 (18.5%)	21 (20.0%)	27 (17.4%)	0 (0.0%)	48 (82.8%)
Heart disease	77 (29.6%)	35 (33.3%)	42 (27.1%)	51 (25.2%)	26 (44.8%)
Diabetes mellitus	47 (18.1%)	18 (17.1%)	29 (18.7%)	38 (18.8%)	9 (15.5%)
Collagen disease	35 (13.5%)	13 (12.4%)	22 (14.2%)	23 (11.4%)	12 (20.7%)
Chronic respiratory disease	30 (11.5%)	14 (13.3%)	16 (10.3%)	23 (11.4%)	7 (12.1%)
Chronic hepatic dysfunction	13 (5.0%)	6 (5.7%)	7 (4.5%)	10 (5.0%)	3 (5.2%)
Malignant tumor	119 (45.8%)	33 (31.4%)	86 (55.5%)	94 (46.5%)	25 (43.1%)
Total parenteral nutrition	42 (16.2%)	19 (18.1%)	23 (14.8%)	33 (16.3%)	9 (15.5%)
Serum albumin < 2.5 g/dL	79 (30.4%)	31 (29.5%)	48 (31.0%)	65 (32.2%)	14 (24.1%)
Surgery within 28 days	105 (40.4%)	36 (34.3%)	69 (44.5%)	83 (41.1%)	22 (37.9%)
Ventilator use	54 (20.8%)	48 (45.7%)	6 (3.9%)	42 (20.8%)	12 (20.7%)
ICU stay	45 (17.3%)	36 (34.3%)	9 (5.8%)	34 (16.8%)	11 (19.0%)
APACHE II score > 10	92 (35.4%)	60 (57.1%)	32 (20.6%)	72 (35.6%)	20 (34.5%)
Steroid use	50 (19.2%)	20 (19.0%)	30 (19.4%)	34 (16.8%)	16 (27.6%)
Immunosuppressive therapy	9 (3.5%)	5 (4.8%)	4 (2.6%)	6 (3.0%)	3 (5.2%)
Anticancer therapy	27 (10.4%)	9 (8.6%)	18 (11.6%)	22 (10.9%)	5 (8.6%)
Bloodstream infections	37 (14.2%)	37 (35.2%)	0 (0.0%)	31 (15.3%)	6 (10.3%)
VAP	38 (14.6%)	38 (36.2%)	0 (0.0%)	31 (15.3%)	7 (12.1%)
Bone and joint infections	36 (13.8%)	36 (34.3%)	0 (0.0%)	24 (11.9%)	12 (20.7%)
Central nervous system infections	2 (0.8%)	2 (1.9%)	0 (0.0%)	2 (1.0%)	0 (0.0%)
Skin and soft tissue infections	63 (24.2%)	2 (1.9%) *	61 (39.4%)	53 (26.2%)	10 (17.2%)
Intra-abdominal infections	26 (10.0%)	0 (0.0%)	26 (16.8%)	19 (9.4%)	7 (12.1%)
Urinary tract infections	8 (3.1%)	1 (1.0%) *	7 (4.5%)	4 (2.0%)	4 (6.9%)
Respiratory tract infections except for VAP	68 (26.2%)	4 (3.8%) *	64 (41.3%)	52 (25.7%)	16 (27.6%)
Piperacillin/tazobactam	54 (20.8%)	19 (18.1%)	35 (22.6%)	49 (24.3%)	5 (8.6%)
Non-steroidal anti-inflammatory drugs	55 (21.2%)	22 (21.0%)	33 (21.3%)	42 (20.8%)	13 (22.4%)
Use of contrast medium	36 (13.8%)	8 (7.6%)	28 (18.1%)	22 (10.9%)	14 (24.1%)
Furosemide	26 (10.0%)	14 (13.3%)	12 (7.7%)	15 (7.4%)	11 (19.0%)
Catecholamine	21 (8.1%)	13 (12.4%)	8 (5.2%)	9 (4.5%)	12 (20.7%)
Angiotensin-converting-enzyme inhibitor/Angiotensin II receptor blocker	37 (14.2%)	20 (19.0%)	17 (11.0%)	28 (13.9%)	9 (15.5%)

* Complication of bloodstream infections. eGFR: estimated glomerular filtration rate; ICU: intensive care unit; VAP: ventilator-associated pneumonia.
